# An efficient context-aware screening system for Alzheimer's disease based on neuropsychology test

**DOI:** 10.1038/s41598-021-97642-4

**Published:** 2021-09-17

**Authors:** Austin Cheng-Yun Tsai, Sheng-Yi Hong, Li-Hung Yao, Wei-Der Chang, Li-Chen Fu, Yu-Ling Chang

**Affiliations:** 1 Department of Computer Science and Information Engineering, National Taiwan University, Taipei, Taiwan; 2 Department of Psychology, National Taiwan University, Taipei, Taiwan

**Keywords:** Dementia, Alzheimer's disease

## Abstract

Alzheimer's disease (AD) and other dementias have become the fifth leading cause of death worldwide. Accurate early detection of the disease and its precursor, Mild Cognitive Impairment (MCI), is crucial to alleviate the burden on the healthcare system. While most of the existing work in the literature applied neural networks directly together with several data pre-processing techniques, we proposed in this paper a screening system that is to perform classification based on automatic processing of the transcripts of speeches from the subjects undertaking a neuropsychological test. Our system is also shown applicable to different datasets and languages, suggesting that our system holds a high potential to be deployed widely in hospitals across regions. We conducted comprehensive experiments on two different languages datasets, the Pitt dataset and the NTUHV dataset, to validate our study. The results showed that our proposed system significantly outperformed the previous works on both datasets, with the score of the area under the receiver operating characteristic curve (AUROC) of classifying AD and healthy control (HC) being as high as 0.92 on the Pitt dataset and 0.97 on the NTUHV dataset. The performance on classifying MCI and HC remained promising, with the AUROC being 0.83 on the Pitt dataset and 0.88 on the NTUHV dataset.

## Introduction

Dementia is one of the most common types of neurodegenerative disorders related to cognitive impairment^1,2^. According to the latest statistics published by the World Health Organization, it is estimated that 50 million people worldwide suffer from dementia, accounting for 5% of the elderly population, and it is expected to exceed 150 million in the next 30 years. It is so severe globally that it has become the fifth cause of death worldwide, and the medical costs are approximately 1.1% of global gross domestic product (GDP)^3–6^, about US$ 818 billion. The increase in the number of patients has placed a heavy burden on healthcare systems, resulting in delayed diagnosis. Among different types of dementia, the Alzheimer's Disease (AD) would be our focus in this study because it is the most prominent and accounts for 60–80% of dementia cases.

The current research divides the progression of AD into three stages: preclinical AD, Mild Cognitive Impairment (MCI) due to AD, and dementia due to AD^5^. In the stage of preclinical AD, some measurable changes can be found in the brain, such as cerebrospinal fluid, or blood, which are the earliest biological markers of AD. It is worth mentioning that not all individuals with these biological signs develop to have MCI or dementia in the future. No apparent cognitive symptoms, such as memory loss, can be observed from individuals at this stage. In the stage of MCI due to AD, mild cognitive declines can be observed and may be noticeable to family members. However, such cognitive difficulties might not significantly impact individuals' ability to handle everyday activities.

In the stage of dementia due to AD, compared with normal age-related change, significant cognitive signs can be observed in patients with AD, including memory loss that disrupts daily life, the challenges in planning or solving problems, the disorientation to place or time information, having difficulty in completing familiar tasks at home, having trouble understanding complex instructions or spatial relationships, losing semantic knowledge^7^.

The diagnosis of AD typically consists of a variety of approaches and tools, including obtaining a medical history from individuals, clinical examination, laboratory assessment, and neuropsychological evaluation^8^, according to the criteria established by the National Institute of Neurological and Communicative Disorders and Stroke (NINCDS) and the Alzheimer's Disease and Related Disorder Association (ADRDA). The picture description test^9–11^ and the logical memory test^12–14^ are two widely used tests in both clinical and research settings among a variety of neuropsychological tests. The picture description test is used to evaluate the individual's discourse ability, which is a part of the language function. During the picture description test, the patient is asked to describe a given picture as detailed as possible. On the other hand, the logical memory test is used to evaluate a person's episodic memory. During the test, two stories are read aloud to an individual, and then the individual is asked to recall the story as precisely as possible.

Traditionally, these tests have to be done under the medical experts' supervision, which requires many human efforts and typically takes a long time. Hence, a tool that can assist the diagnosis process is imperative. Existing literature mostly heavily depends on manual feature pre-processing and follows the below procedure. First, an excessive number of features are manually generated. The features are then selected and used to train a classifier, such as Support Vector Machine (SVM) or Random Forest. For example, Fraser et al.^15^ extracted 370 features, including part-of-speech, syntactic complexity, vocabulary richness, and repetitiveness, to capture a wide range of linguistic characteristics. Hernández-Domínguez et al.^16^ constructed a referent from healthy participants and evaluated the data using coverage measures between speech and the referent. A total of 105 features were used in their research, including information coverage, linguistic and speech features. On the other hand, Yancheva et al.^17^ focused on topic modeling, and they converted noun and verb words in the transcripts to a vector space representation using GloVe^18^ word vectors. They further extracted 12 features, including distance measures, idea density, and idea efficiency, to train a classifier accordingly.

The studies mentioned above have demonstrated high performance and high potential automatic usage. However, these studies have several drawbacks. First, manual pre-processing was required before the features were obtained, including word replacement (e.g., changing "brother" to "boy") and lemmatization (e.g., changing "running" to "run"). Second, the extracted features of these studies might depend on the syntactic characteristics of the data. Because patients with AD may experience verbal disfluencies during data collection, it is difficult to get precise identifiers from the existing tools. Third, some studies achieved a higher performance by using a feature selection step, which was inefficient for an automatic system. Fourth, most of these studies focused on examining only a specific language. It would be crucial to develop one screening system that could be generalized and applied to different languages.

Therefore, in this study, we proposed a novel automatic screening system capable of distinguishing subjects with early AD from HC and distinguishing subjects with MCI from HC with different languages. The picture description test and the logical memory test are selected in our system because they have been shown to be sensitive to identifying patients with AD. The collection of speech information is relatively undemanding than other materials. We designed a novel deep-learning model in the system to carry out the classification. The proposed system has superior efficiency over previous works while reducing the pre-processing efforts on the data. Moreover, it is generalizable to the speeches made in various languages and could be easily implemented in a hospital setting owing to its low computational costs. Finally, our model requires no pre-defined syntactic features and the feature selection step. The model would automatically learn to discriminate important features during the training phase, hence reducing medical professionals' time and efforts. We evaluated our deep-learning model with a tenfold cross-validation process with five runs to obtain an AUROC of classifying AD and healthy control (HC) of 0.92 on the Pitt dataset and an AUROC of 0.97 on the NTUHV dataset. For the classification between MCI and HC, the performance remained promising, with the AUROC being 0.83 on the Pitt dataset and 0.88 on the NTUHV dataset.

## Methods

### Neuropsychological tests and data collection

The present study was approved by the Ethics Committee and Institutional Review Board at National Taiwan University Hospital, and all the experiments were performed in accordance with the guidelines and the regulations in the National Taiwan University Hospital. The written informed consent was obtained from all participants. Two different neuropsychological tests were used in this study.

1. Logical memory test

During the logical memory test, two stories were read loudly to the subject, and the subject was asked to recall the story as much as possible after each read of the two stories. To minimize the burden on the subject and to reduce the screening time of the system, only data of the immediate recall of the first story was used for developing our system. The total screening time was less than three minutes. The story used in this work was part of the subtest of the Wechsler Memory Scale III (WMS-III)^19^. The story was presented in Mandarin Chinese.

(2) Picture description test

During the test, a picture was presented to the subject, and the subject was asked to describe what he or she saw as detailed as possible. The response time was set to be one-minute long in the present study, and the total administration time was less than three minutes. The picture used in this work was chosen from the Western Aphasia Battery (WAB)^20^.

### Data collection procedure

Data collection for the two tests was conducted using the following procedure: First, the subject was asked to sit in front of the desk holding a microphone. Second, the instruction of the task was read out to the subject, and then the subject was requested to respond based on the test instructions. Overall, it took less than five minutes to finish the two tests.

For the logical memory test, the instruction for the test was, "I will read a short story to you, please pay attention, and try to remember all the content and details of the story. I want you to recall the story the way I read it to you as precisely as possible. Ready?", which was followed by the story. For the picture description test, the instruction used in data collection was "Next, please take a look at this picture. I want you to describe this picture as detailed as possible within one minute. Are you ready?"

### Dataset acquisition

In this study, two different datasets were used to evaluate the performance of the proposed system, namely, the Pitt Dataset^21^ and the NTUHV Dataset. The Pitt Dataset is an open dataset that collects transcripts and audio files from the speeches of several hundred healthy subjects and subjects with AD or MCI living in the United States while undertaking the picture description test. The NTUHV Dataset is the private dataset collected in the present study. It contains the transcripts and audio files from the speeches made by healthy subjects and subjects with AD or MCI living in Taiwan during the test following the data collection procedure as mentioned above. Here, all the subjects recruited through a large ongoing aging cohort study for both tests were diagnosed to either AD, MCI, or HC by the psychology specialists, and their corresponding transcript data we annotated with suitable labels (AD/MCI/HC) accordingly. In particular, AD diagnosis was based on a set of clinical diagnostic criteria developed by the NINCDS-ADRDA^22^. In contrast, classification as MCI or as HC was based on cognitive tests excluding searching biomarkers.

To properly train our deep learning model on both datasets, we keep the number of data in every class identical to prevent data imbalance and learning bias. Detailed information for the selected subjects (AD/MCI/HC) of the Pitt dataset and the NTUHV dataset can be found in Tables 1, 2, 3, and 4.

**Table 1:** Subject information of the Pitt dataset (AD vs. HC).

	AD	HC
Number of subjects	257	242
Male	87	88
Female	170	154
Age	71.6 ± 8.3	64.4 ± 7.6
Years of education	12.3 ± 2.8	14.0 ± 2.4
MMSE	18.8 ± 5.0	29.1 ± 1.0

**Table 2:** Subject information of the Pitt dataset (MCI vs. HC).

	MCI	HC
Number of subjects	43	43
Male	27	13
Female	16	30
Age	69.7 ± 7.2	67.0 ± 6.4
Years of education	14.9 ± 2.6	14.0 ± 2.1
MMSE	27.4 ± 1.6	29.1 ± 0.9

**Table 3:** Subject information of the NTUHV dataset (AD vs. HC).

	AD	HC
Number of subjects	40	40
Male	16	18
Female	24	22
Age	77.6 ± 7.4	69.1 ± 7.2
Years of education	12.7 ± 3.5	15.1 ± 3.3
MMSE	21.8 ± 3.4	28.6 ± 1.4

**Table 4:** Subject information of the NTUHV dataset (MCI vs. HC).

	MCI	HC
Number of subjects	30	30
Male	14	11
Female	16	19
Age	74.6 ± 6.5	68.4 ± 7.6
Years of education	14.2 ± 4.4	15.0 ± 3.3
MMSE	26 ± 2.6	28.9 ± 1.5

### Proposed deep-learning model

In this study, we aim to predict the probability of the participant being in the AD group or HC group based on the speech materials. We formulated our problem into a text classification problem, in which a sequence of words from a subject was used to classify the subject into a particular class (AD or HC). The problem formulation could hence be written as:

1 $$f\left( X \right) = p(having \,AD|X)$$

2 $$X = \left[ {x^{\left( 1 \right)} ,x^{\left( 2 \right)} , \ldots ,x^{\left( T \right)} } \right]$$

where $x^{\left( i \right)} , i \in \left[ {1, T} \right],$ denotes the $i$-th word in the spoken speech, $T$ is the length of the speech, and $f\left(X\right)$ is the function that maps $X$ into the probability of being in the AD group.

**Figure 1:**
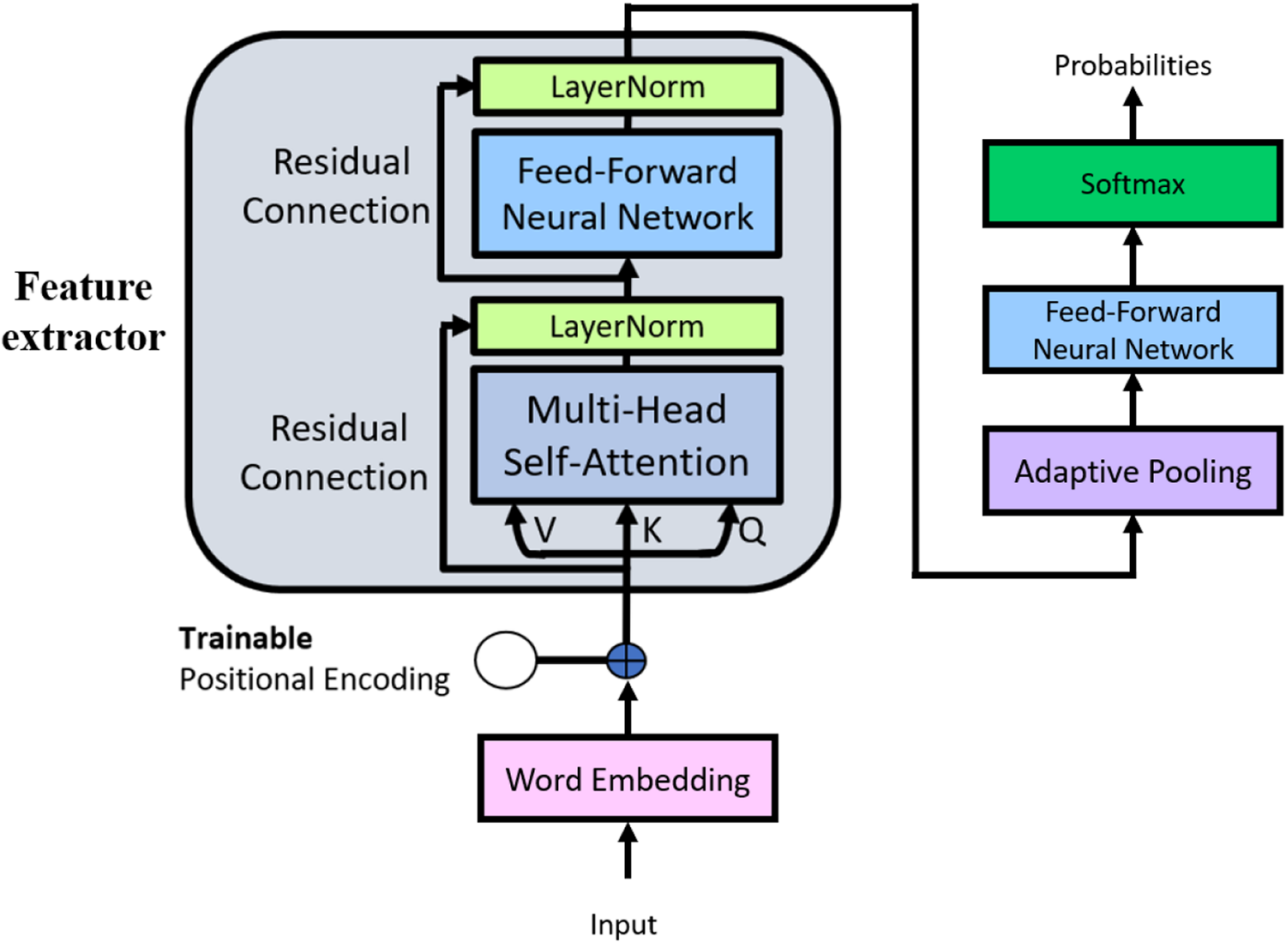
Overview of the proposed deep-learning-based model.

We proposed a novel deep-learning-based model with fast speed and high performance as the mapping function to achieve the end-to-end mapping function $f\left(X\right)$. The overall architecture is illustrated in Fig. 1. In this section, we outlined several critical designs in the model architecture, all of which significantly boost the performance of the system.

#### Embeddings

The embedding layer was used to transform every word into its corresponding word vector. The rationale behind this transformation was to map each word into a high-dimensional space to represent the word with a high-dimensional word vector that contained semantically meaning of the word and enabled further comparison and computation between words. Word vectors could be pre-trained on general language corpus such as Wikipedia to capture the general semantic meaning and the relationship between words of the language. The pre-trained word vectors could provide better representation for each word and offer better semantic meaning than the randomly chosen word vectors.

#### Self-attention

The self-attention mechanism extracted inter-sequence (inter-sentence in our case) relationship and eliminated the long-term-dependency problem of traditional recurrent-neural-network-based models, which have long been a widely-used technique in Natural Language Processing^23^. In the self-attention mechanism or self-attention layer, every word vector in a sentence was "compared" with all the other word vectors. The "comparison" was a function that gave each and every other word a weight, representing its similarity of importance compared to the current word. The final output of the current word vector passing through the self-attention layer was the weighted sum of all the word vectors in the same input sentence, where the weight assigned to each word vector was the result of the comparison function. In the present study, we used the scaled dot-product as the comparison function. Formally, denoting the length of the inputted sequence as $l$, to compute the result for $i$-th word with word vector ${v}_{i}$, we used every other word vector ${v}_{j}$ as the query vectors and current word vector ${v}_{i}$ as the key vector to compute a weight $\left({v}_{j}^{T}{v}_{i}\right)$. The transformed vector ${v}_{i}^{{\prime}}$ for the $i$-th word was then the weighted sum of ${v}_{j}$ for every position $j$ in the inputted sequence:

$$v_{i}^{{\prime}} = \mathop \sum \limits_{j = 0}^{l} \left( {v_{j}^{T} v_{i} } \right)v_{j}$$

#### Multi-head attention

The concept of multi-head attention was to apply a self-attention mechanism several times with different transformation matrices, which was referred to as "heads" in this study. Using multiple heads was helpful especially when the text was not fully informative and the text from different perspectives, such as syntactic or semantic aspect, needed to be viewed as indicated by Jawahar et al*.*^24^*.* The results of different heads are concatenated and use a fully-connected layer to restore the dimension to the original one. Formally, denoting the length of the inputted sequence as $l$, to compute the result for one single head, every word vector was transformed with different matrices $Q$, $K$, $V$ to form query (${q}_{j}$), key (${k}_{j}$), and value (${p}_{j}$) vectors, respectively. Mathematically,

$$q_{j} = Qv_{j} , k_{j} = Kv_{j} , p_{j} = Vv_{j} , \forall j \in \left[ {0,l} \right]$$

The transformed vectors ${v}_{i}^{{\prime}}$ for this head (Q, K, V) then become

$$v_{i}^{{\prime}} = \mathop \sum \limits_{j = 0}^{l} \frac{{\left( {q_{j}^{T} k_{i} } \right)}}{\sqrt n }p_{j}$$

where $n$ is the dimension of the key vector. Dividing by $\sqrt{n}$ prevents the model from generating an extremely small gradient in later computation (Softmax function), thus making the training of the model more stable. More details about this design can be found in the original paper^20^.

#### Adaptive pooling layer

After passing through the multi-head attention, each word vector interacted with every other word vector, captured subtle information within the given sentence, and finally produced a transformed vector. After the transformed vectors were computed in the sentence, they were sent through an adaptive pooling layer to automatically summarize all the word vectors in the whole sentence into a single vector, the sentence embedding, which contains the predictive information of the whole sentence for later classification.

#### Overall model

The overall model was composed of several basic feature-extraction blocks. The input words are firstly transformed into high-dimensional word vectors. Then, the feature-extraction block used the multi-head attention and feed-forward neural network to extract necessary features from the input word vectors. Residual connection and layer normalization were used to enhance the performance of the model. After inputs were processed through *N* feature-extraction blocks, a pooling layer was used to summarize the features of every word into a single sentence feature, and a non-linear classifier was used to transform the sentence feature into the probability for each class.

### Proposed screening engine

**Figure 2:**
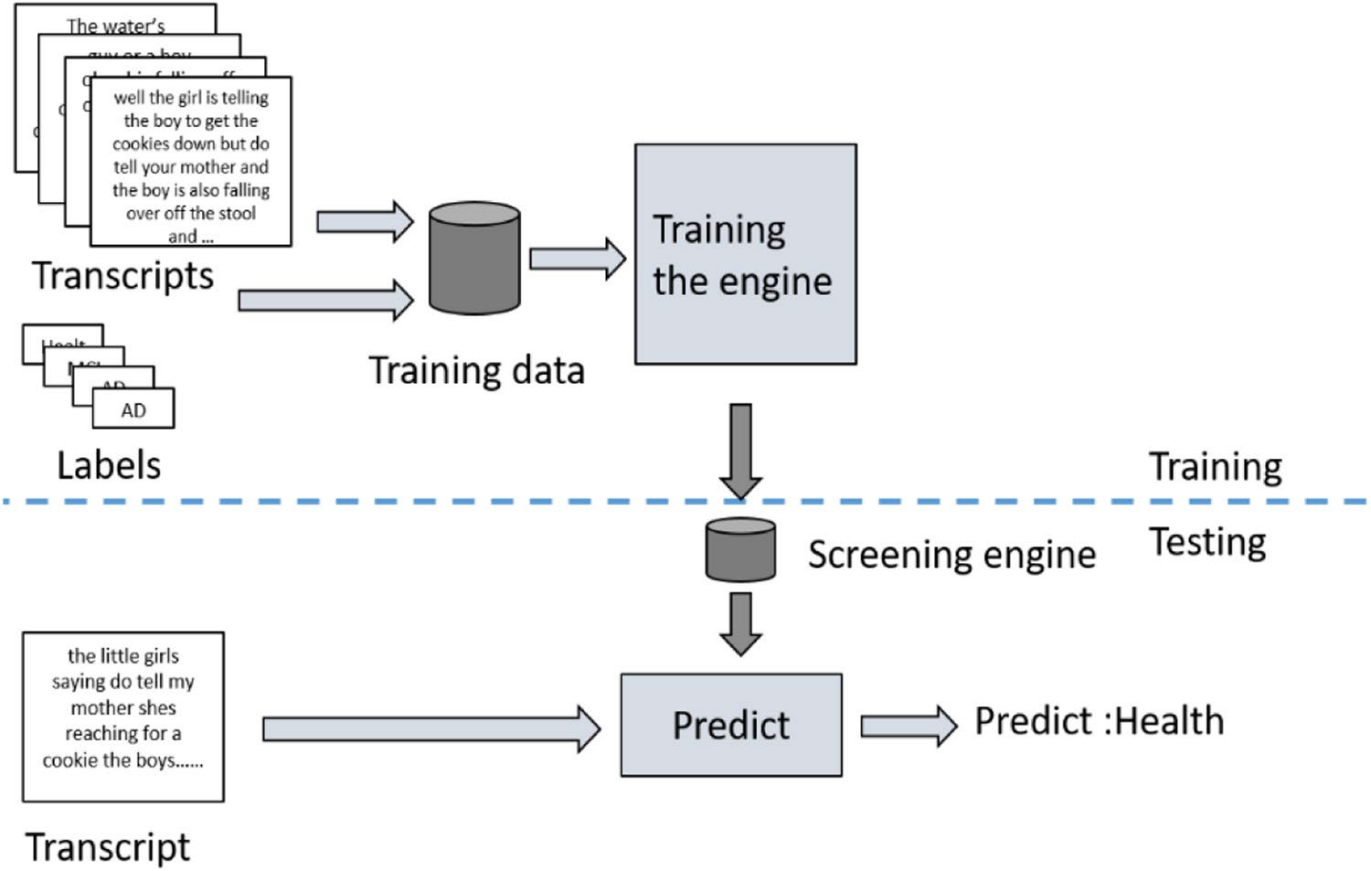
Overview of the proposed screening engine.

An overview of the system of the screening engines is shown in Fig. 2. There were three screening engines used in this work, which were the model using data of the picture description test, the model using data of the logical memory test, and the model using both types of data. A series of experiments using different tests were conducted to examine their performance.

1. Engine with single neuropsychological test

**Figure 3:**
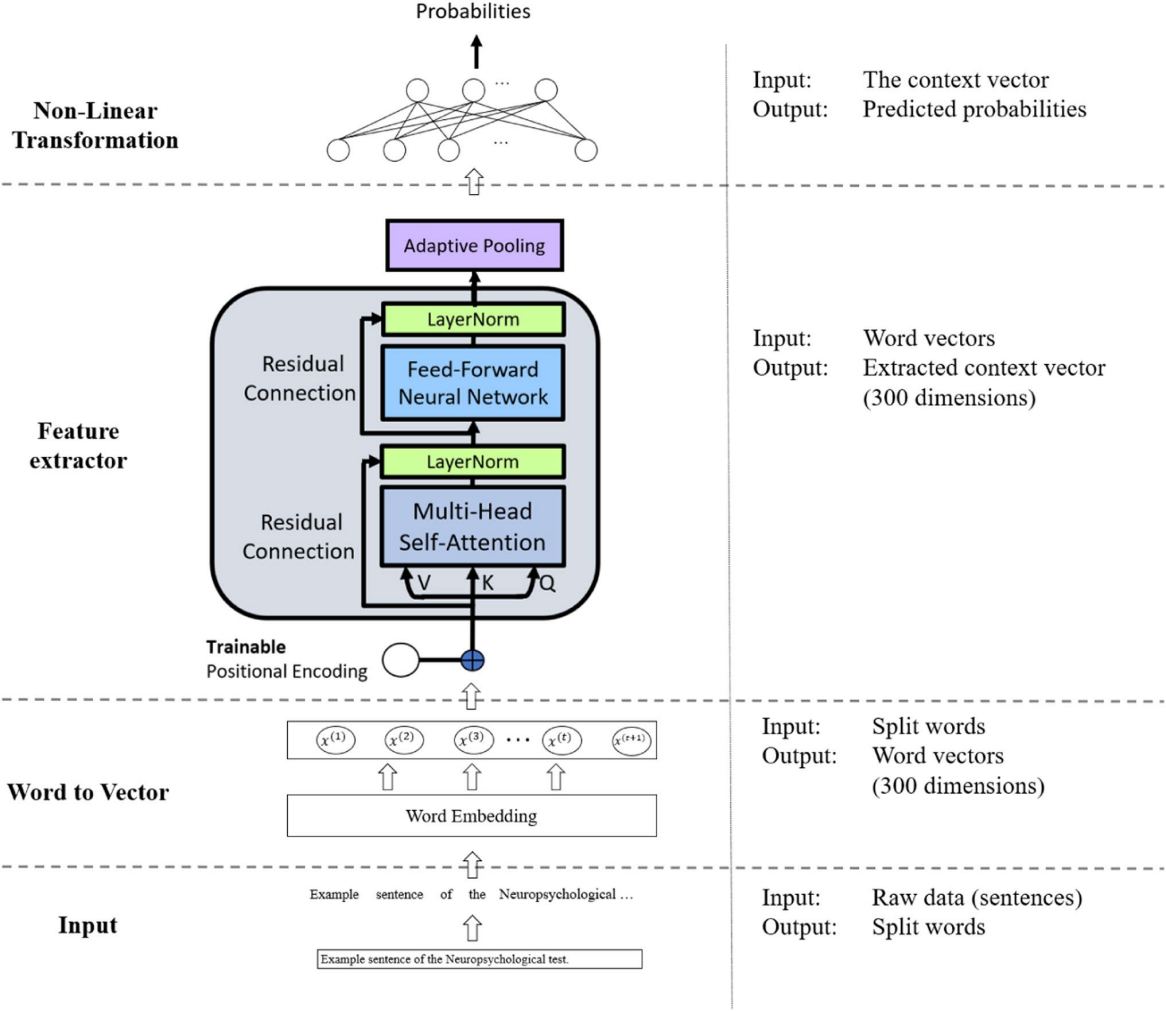
Screening engine using a single neuropsychological test.

For the screening engine used for a single neuropsychological test, the network architecture is depicted in Fig. 3. The model is constructed on the basis of the techniques mentioned in Sect. 2.3. The input of the system is the verbal text data generated by subjects. The text data were then sequentially processed by an embedding layer, a feature extractor, and finally, a non-linear transformation, all of which were performed automatically in an end-to-end fashion. The output was the probability of being classified as AD.

Each word in the transcript was firstly fed into the embedding layer in order to be converted into the corresponding word vectors. Here, we used the pre-trained word vectors fasttext^25^, since it was one of the most semantic-rich word vectors, and it is also multilingual, with a total of 157 different languages available. To assess the flexibility, we tested different complexity of the fasttext word vectors in the model in the *Result* section. After converting words to word vectors, they were inputted to a feature-extraction block (N=1), with four heads for multi-head self-attention.

The idea of using multiple attention networks was to force the model to learn the information units, which was a set of informative words that could be used to distinguish AD/MCI and HC. The attention mechanism has been successful in many NLP tasks that need to highlight the informative words and deduct relationships in the transcript. Since the transcript might consist of several kinds of information units, a single attention weight would be incapable of finding all the important units in the transcript. For example, Croisile *et al.*^9^ defined four categories and 36 information units on the cookie theft picture to evaluate the performance of the subject undertaking the test. Similar scenarios could be found in the logical memory test as Johnson *et al.*^26^ listed 35 key units in their analysis. For these reasons, one single feature-extraction block with four heads (multi-head) attention network was used in the present study.

The feature-extraction block allowed information to pass between words and to extract important information from the whole sentence. The block produces feature vectors for each word, and the vectors were passed into a sentence aggregator. In this study, we used max pooling, average pooling, and bi-directional Gated Recurrent Unit^27^ (Bi-GRU) together as the pooling layer. Max pooling and average pooling extracted important information within a sentence, while the Bi-GRU was used to ensure the sequential relationship between words. Finally, the sentence vector was transformed with a fully-connected layer with ELUs^28^ as activation functions and a Softmax layer to convert to probability.

(2) Engine with two neuropsychological tests

**Figure 4:**
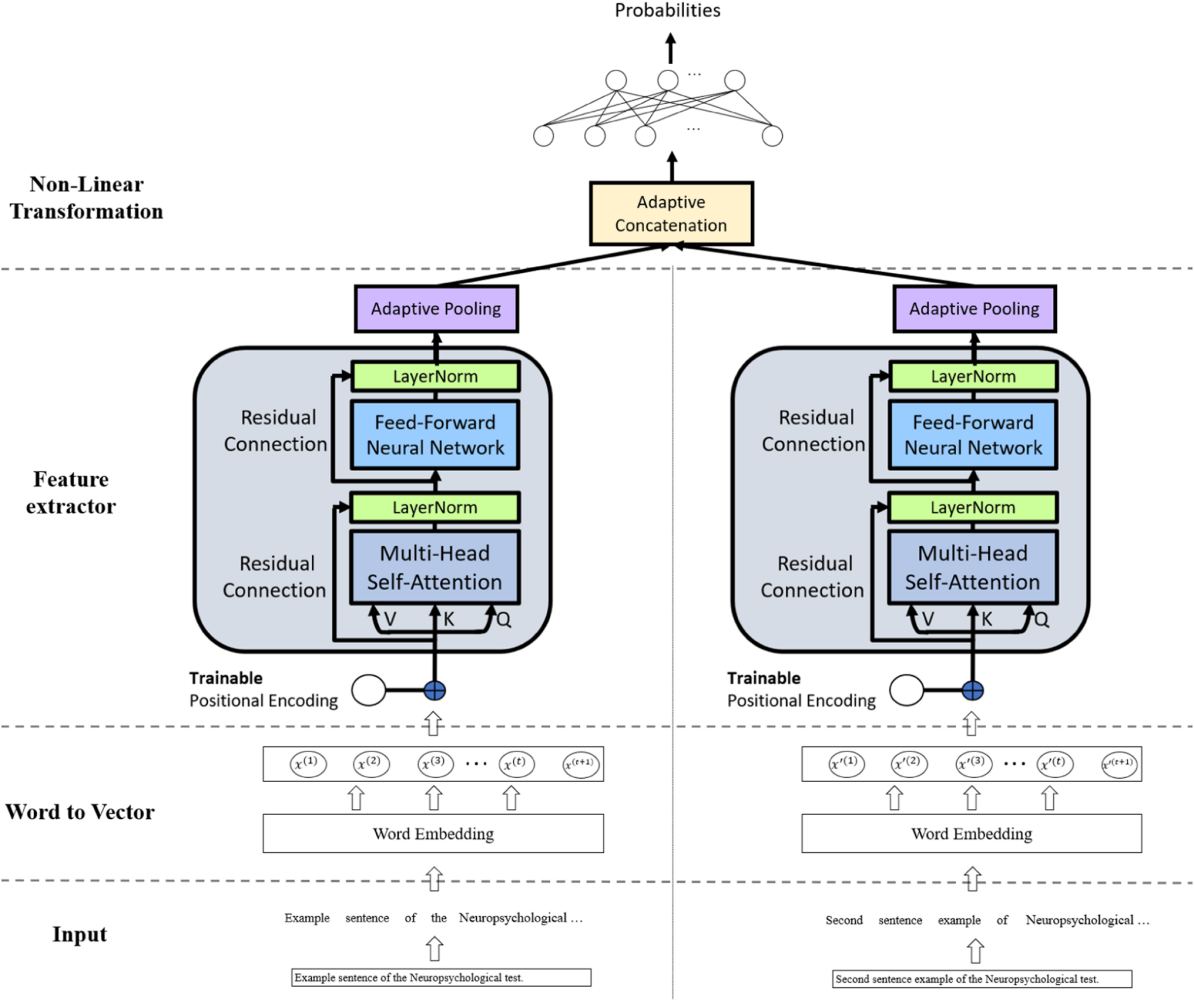
Screening engine using two neuropsychological tests.

Figure 4 shows the architecture of the screening engine for the two neuropsychological tests. To better leverage characteristics of different tests, the model was formed by merging two models of a single test. These two models were first trained individually on data from one of the two tests, and their parameters were optimized based on their respective losses. After the training of two models, the output layers of both models were removed, and the last fully connected layer before the output layer of the two models was concatenated and followed by a new output layer, which is a single layer feed-forward neural network. The merged model was then fine-tuned on both datasets. All the parameters remained trainable during the training stage. The merged model outputted the probability of the subject being classified in the AD group.

(3) Model training

The model was trained with a learning rate of 0.0001 using Adam^29^ as the optimizer. For the hyper-parameters in the Adam optimizer, ${\beta }_{1}$ was set as 0.9, ${\beta }_{2}$ was set as 0.999, ϵ was set as ${10}^{-8},$ and γ was set as ${10}^{-4}$. The batch size was set as 32, and the number of iterations was set as 80. For the model for both tests, two models for a single test were merged, and were fine-tuned with data from both tests using the same hyperparameters, and were trained with 150 iterations.

The model was trained by minimizing the cross entropy between the prediction and the ground truth, which, in our scenario, is to maximize the probability of the correct class and minimize the probability of the wrong class. This could be formally written as:

3 $$loss = - \mathop \sum \limits_{i} y_{i} \log \left( {\hat{y}_{i} } \right)$$

where ${\widehat{y}}_{i}$ was the prediction of the model, and ${y}_{i}$ was the one-hot label of the training data. This formulation also enables the model to be trained in a batched fashion, leading to faster training speed (less inference time) and more accurate results.

## Results

### Evaluation of the screening engine

In the next section, we conducted extensive experiments to validate our model. Unless otherwise specified, all metrics except AUROC are reported using a threshold of 0.5, i.e., Predicted probability larger than 50% would be classified as AD (positive class), whereas probability smaller than 50% would be deemed as HC (negative class). Even though accuracy can be tuned higher for about 1–5% if we do not use a threshold of 0.5, we still used 0.5 as the threshold for fairer comparison between datasets and studies in the future, and instead, we use AUROC to present the discriminative ability of the proposed model.

To evaluate the effectiveness of our model, we carefully performed all experiments using five runs of 10-fold cross-validations. First, in every iteration, the data were shuffled randomly and were divided into ten splits. Next, nine splits of data were used as the training set, and the remaining split of the data were used as the test set to assess the performance. The iteration was repeated ten times to ensure that every sample in the dataset was tested. In every iteration, we reset the model and selected one split for testing and used the remaining splits for training. This prevents the “data leak” issue that some training data also appear in the test data, which may yield inflated performance. The completion of the above steps was called a trial. Finally, all results reported in this work were averaged over the five trials to minimize the effect caused by the partition of the training and test set.

### Performance of classifying AD vs. HC

**Figure 5:**
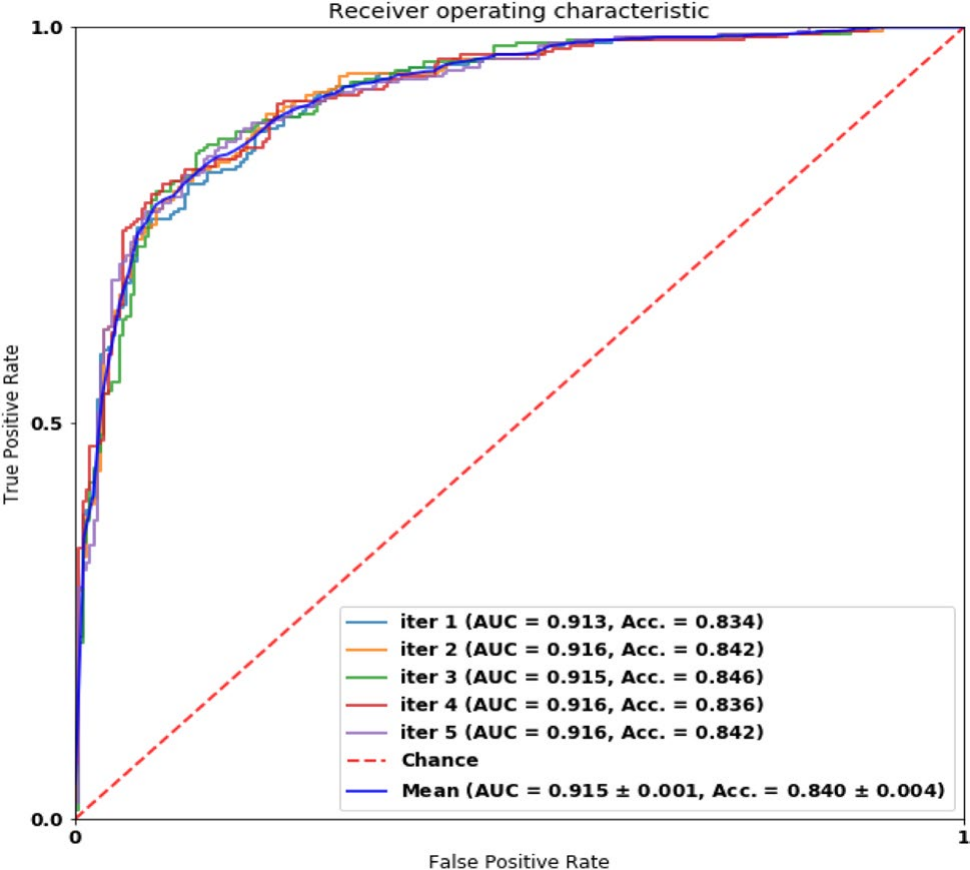
AUROC of classifying AD against HC on the Pitt dataset.

We reported AD vs. HC's classification performance on two datasets, the Pitt dataset and the NTUHV dataset, to evaluate our model's generalization capability to different languages and different ethnicities. On the Pitt dataset, we only have the picture description test, as it contains only the transcript data of the picture description test, whereas we have two tests on the NTUHV dataset. Comprehensive results can be found in Table 5, which shows our model's performance with various metrics commonly used in the field of medical screening systems. The AUROC curve on the Pitt dataset is shown in Fig. 5, indicating the stability (low variance) across different trials and our model's strong predictive power.

**Table 5:** Performance of AD *vs.* HC on different datasets.

Dataset	Sensitivity	Specificity	Accuracy	AUROC
Pitt-pic	0.82 ± 0.01	0.85 ± 0.01	0.84 ± 0.00	0.92 ± 0.00
NTUHV-pic	0.87 ± 0.01	0.84 ± 0.02	0.85 ± 0.01	0.92 ± 0.01
NTUHV-mem	0.88 ± 0.03	0.91 ± 0.01	0.89 ± 0.01	0.96 ± 0.00
NTUHV-both	**0.95** ± 0.02	**0.92** ± 0.02	**0.93** ± 0.01	**0.97** ± 0.01

Bold is used to highlight the highest value in each column.

Our model demonstrated strong performance and stability on all the metrics on both datasets, which indicated the robustness and generalization capability on different countries and languages without compromising the performance. Notably, our proposed model achieved 0.92 in mean AUROC and 0.84 in accuracy on the Pitt dataset if the threshold was set to 0.5. On the NTUHV dataset, the model has a surprisingly higher 0.97 mean AUROC and 0.93 in accuracy. In terms of other criteria, our model achieved 0.82 mean sensitivity, 0.85 mean specificity on the Pitt dataset, whereas achieving 0.95 in mean sensitivity and 0.92 mean specificity on the NTUHV dataset.

It is worth noting that the engine using the logical memory test performed better in all criteria against the results based on the picture description test. This is reasonable because the logical memory tests access a cardinal cognitive characteristic, the episodic memory function, that is most susceptible during the development of AD^30^. The engine using both types of data performed better in all metrics than that of using only one type of data, since the model had more information on different aspects of the individual's cognitive profiles.

Table 6 presents the accuracy of our work compared to that of the previous works. It is indicated that our model significantly outperforms all the previous works with a large margin in terms of accuracy.

**Table 6:** Related works of the performance of AD *vs.* HC on Pitt datasets.

Work	Numbers of subject	Accuracy
Matej et al.^34^	156	0.77
Guerrero-Cristancho et al*.*^33^	196	0.82
Fraser et al*.*^15^	473	0.82
Pompili et al*.*^31^	475	0.77
Yancheva et al*.*^17^	496	0.8
Hernández-Domínguez et al*.*^16^	499	0.79
Ours (Pitt-pic)	499	0.84
Ours (NTUHV-both)	60	0.93

### Performance of classifying MCI vs. HC

**Figure 6:**
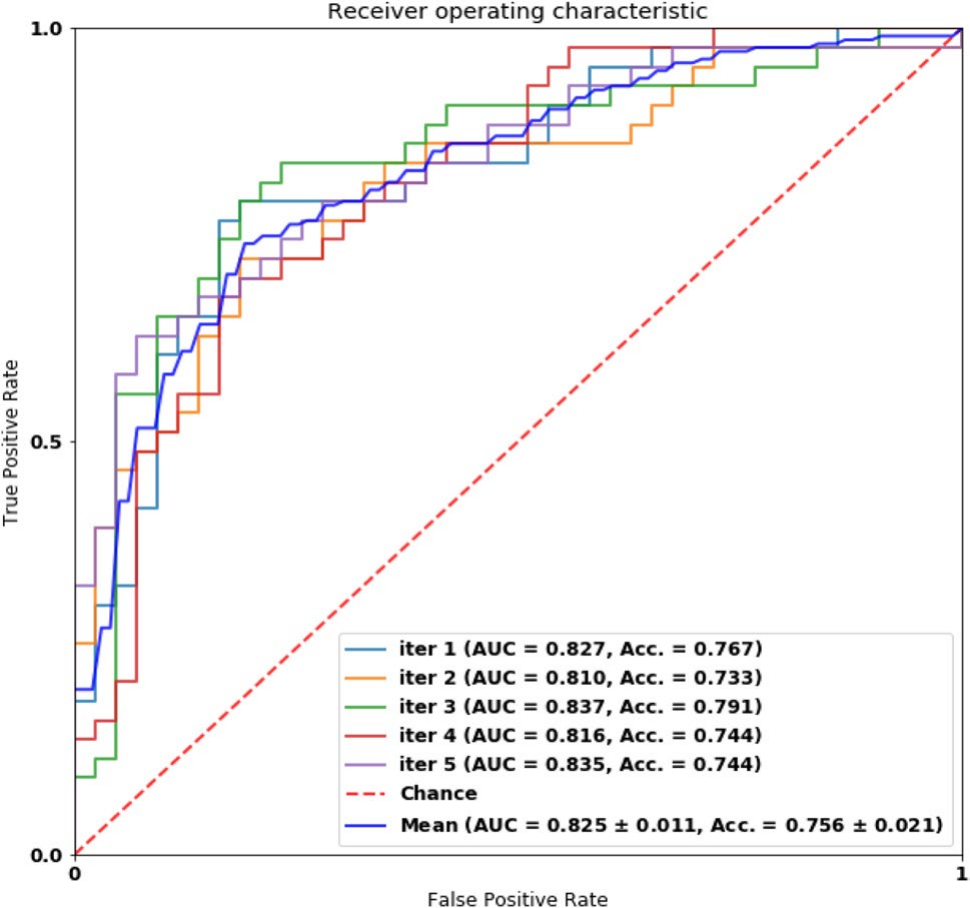
AUROC of classifying MCI against HC on the Pitt dataset.

We report the classification performance of MCI vs. HC on different datasets in Table 7. Our engine achieved 0.83 in AUROC and 0.76 in accuracy on the Pitt dataset, whereas achieving 0.88 and 0.82 in accuracy on the NTUHV dataset. The AUROC curve on the Pitt dataset is shown in Fig. 6.

**Table 7:** Performance of MCI *vs.* HC on different datasets.

	Sensitivity	Specificity	Accuracy	AUROC
Pitt (pic)	0.77 ± 0.03	0.74 ± 0.03	0.76 ± 0.02	0.83 ± 0.01
NTUHV (pic)	0.83 ± 0.02	0.66 ± 0.01	0.75 ± 0.02	0.82 ± 0.01
NTUHV (mem)	0.84 ± 0.03	**0.80** ± 0.03	0.82 ± 0.02	0.86 ± 0.00
NTUHV (both)	**0.91** ± 0.02	0.72 ± 0.02	**0.82** ± 0.02	**0.88** ± 0.02

Bold is used to highlight the highest value in each column.

Our model performed very well on both the Pitt dataset and NTUHV dataset in terms of all the metrics. Results on other criteria of the proposed network are presented in Table 7. The mean sensitivity, mean specificity, and mean accuracy based on our model were 0.77, 0.74, 0.76, respectively, on the Pitt dataset and were 0.91, 0.72, 0.82, respectively, on the NTUHV dataset. The performances in the present study were higher than all the existing works, which was shown in Table 8. It is noteworthy that fewer works were compared as most works focus solely on identifying AD. This also demonstrated our system's strength in that it consistently provides promising results on both classification tasks (AD vs. HC and MCI vs. HC).

**Table 8:** Related works of the performance of MCI *vs.* HC on Pitt datasets.

Work	Numbers of subject	Accuracy	AUROC
Santos et al*.* (Pitt-pic)^32^	86	0.65	–
Orimaye et al*.*^35^	38	–	0.74
Ours (Pitt-pic)	86	0.76	0.83
Ours (NTUHV-both)	60	0.82	0.88

"–"means that the statistics is not reported.

Despite that our work is with superior performance than the previous works, the classification between MCI and HC was less optimal than the classification results between AD and HC on all the datasets. One possible explanation was that, because the cognitive deficits of MCI were subtle, it would be much more challenging to separate MCI from HC. This was also evident that the variance of the performance between different trials was slightly greater, and results were less stable than those in the classification between AD and HC.

### Influence of word vectors

The word vector dimension in the above experiments is set to 300, which can provide the most linguistic information compared to that of using lower dimensions. This section investigates the impact of using lower dimension word vectors to testify whether our model can work in a low-resource setting as the lower dimension of word vectors implies less computation and less computation time.

1. Pitt dataset

**Figure 7:**
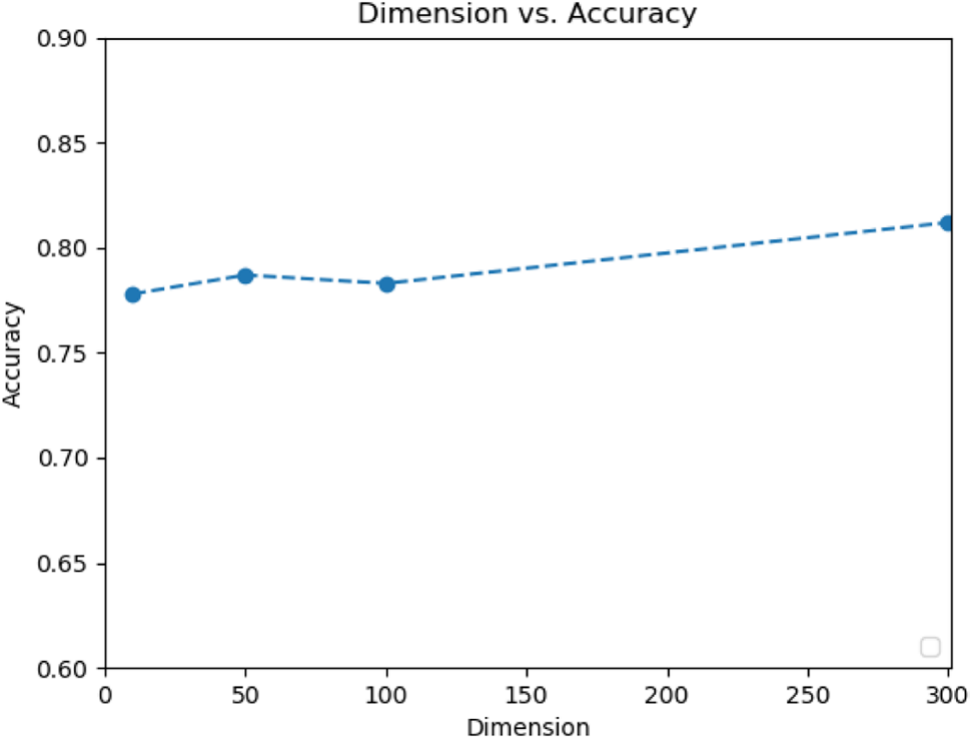
Effect of the dimension of word vectors on the Pitt dataset.

Figure 7 shows the performance of the model using different dimensions of fasttext word vectors. The best result was achieved by using word vectors with 300 dimensions. The accuracy was 0.77 even when the word vectors were reduced to 10 dimensions. The result showed that the proposed model was robust under different settings and was not influenced significantly by the word vectors' dimension. Moreover, it also showed that, even if the high-quality word vectors were not available, one could train their small word vectors to obtain a promising result, as the results of using only 10-dimension word vectors are still competitive.

(2) NTUHV dataset

**Figure 8:**
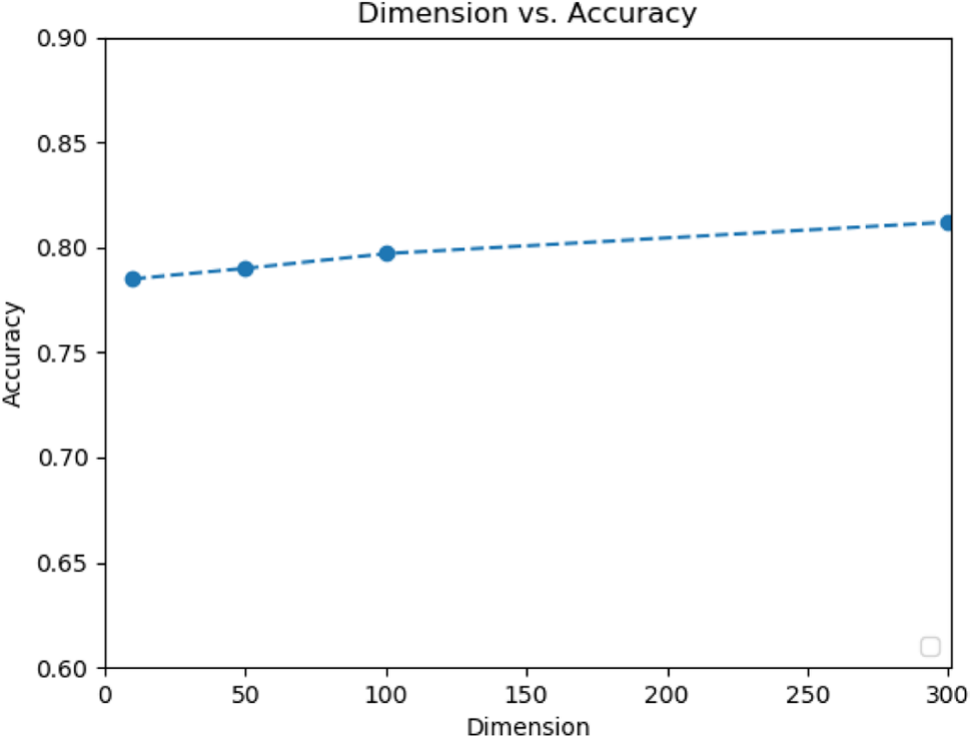
Effect of the dimension of word vectors on the NTUHV dataset.

Figure 8 shows the model's performance using the data of the picture description test and different dimensions of fasttext word vectors. The best result was achieved when word vectors were with 300 dimensions, whereas the worst performance was observed when the word vectors were with only ten dimensions, where the accuracy still achieved 0.79. Complying with the results in the Pitt dataset, the result revealed that the model remained a high performance even when the information provided by the word vectors was limited.

## Discussion

The proposed work has a highly promising performance on classifying elders with cognitive impairment from the cognitively unimpaired elders with significantly fewer constraints on the data collected. Our proposed system is easy to implement, and the model can be used with much less assistance from the psychology specialists, which makes the system more suitable to be used in the clinical setting. Furthermore, the proposed model can be applied to both English and Chinese environments. Since the word vectors in our design, fasttext, provides 157 languages, our system apparently has a high potential to be generalizable to different adopting different languages. It is also noteworthy that the results on classifying AD and HC performed better on our local NTUHV dataset, better than the Pitt dataset collected in USA, mainly because our model can admit additional logical memory test data, which together with picture description test data forming a hybrid data input begin more informative.

More specifically for this hybrid type of data input, for classifying AD and HC in particular, the result demonstrated that using both neuropsychological tests could greatly improve the performance. This is expected because the two tests access different aspects of cognitive functions and could provide a more comprehensive evaluation than a single test. However, the performance on classifying MCI and HC using both neuropsychological tests appears comparable to the same classification task but using only the logical memory test. This might be because other intact cognitive abilities could compensate the performance on the picture description test (e.g., reasoning, semantic knowledge) for individuals with MCI. Such cognitive symptoms of MCI are subtle compared to healthy subjects. Several previous studies also evidenced this point as they found that using the cookie theft picture might render lower classification performance as compared with using a more complicated picture. In this study, we simply use the cookie theft picture in the test to compare the performances of classification on subjects from different countries. Future studies may consider using a more complex picture in order to improve the classification performance.

The screening system was based on multi-attention layers with residual connections, layer normalization, and adaptive pooling techniques. Word vectors were used to provide the semantic and syntactic features of each word. Although the previous studies have used the extracted syntactic and semantic features heavily relying on the feature selection process, the hereby proposed method was completely data-driven and did not require any procedure of pre-processing the data, thus reducing professionals' efforts.

Despite our novel contributions, some limitations should be noted. First, the transcripts used in this work were manually transcribed from the audio recording. In our future work, such a manual way would be replaced by that using Automatic Speech Recognition (ASR). Moreover, our future research intends to use more challenging cognitive tests to achieve better performance, particularly for the classification of MCI and HC. Second, the experiment showed that the average ages of AD subjects and healthy subjects were different though, our research results tended to remain certain predictability once their years of education were over certain degree. But to have true confidence on this predictability, we will need to conduct more study on more consistent age groups in AD cohort *vs*. HC cohort and MCI cohort *vs*. HC cohort in our future research. Moreover, the prediction results were purely based on the input text, and the effect of the mismatch in age can be alleviated as the text contains almost no individual identities. In the future, we will collect more data to make the age distribution closer between groups for fairer comparisons and extend our method to other contextual data without requiring the individual to pass a specific test.
